# Maximizing Access and Minimizing Barriers to Research in Low- and Middle-Income Countries: Open Access and Health Equity

**DOI:** 10.1007/s00223-023-01151-7

**Published:** 2023-11-14

**Authors:** Haroon Saloojee, John M. Pettifor

**Affiliations:** Department of Paediatrics and Child Health, Faculty of Health Sciences, University of the Witwatersrand, Johannesburg, South Africa

**Keywords:** Open access, Article processing charges, Research publications, Journals, Subscription-based journals

## Abstract

Access to published research has always been difficult for researchers and clinicians in low- and middle-income countries, because of the cost of and lack of access to the relevant publications. The dramatic recent increase in electronic research publications has resulted in a marked improvement in reader access to these publications through their mainly Open Access policies, however the costs of processing of submissions and publication have now become the burden of the researchers wishing to publish, rather than the readers. For many researchers working in LMIC, the Article Processing Charges (APC) are prohibitive, hampering the publication of research being conducted in and relevant to these countries. A number of grant funding agencies and international not-for-profit organizations are trying to address these issues by including funding for article publications in their grants, or by supporting publishing entities by subsiding the cost of publication, but more needs to be done by major journal publishers through markedly reducing the APC being charged to researchers in LMIC for open access facilities.

Building research capacity and improving healthcare in less resourced settings depend on students, clinicians, researchers, and scientists being able to read and publish scientific articles. Open access (OA) publications have been hailed as a transformative model for scientific communication, promising unrestricted access to research findings, replacing the traditional subscription model. Approximately a third of global research articles are now being published as OA, and there is a strong drive to further increase this number [1]. Currently, over 13,250 OA journals without article processing charges (APCs) are registered in the Directory of Open Access Journals [2].

The move to OA publication has shifted the cost of publication from the reader (through subscriptions) to the author(s) of research articles. Thus, somewhat paradoxically, in less resourced settings, easier access to scientific publications also means fewer opportunities for researchers to publish and share their findings because of what is viewed to be exorbitant APCs or publication fees, unless they publish in subscription journals, where much of the costs are borne by the journal. The median cost for publishing an OA article in a medical journal is about $4600 [3], and costs range from $2000 to $12,000 (the APC of Calcified Tissue International is $3590). Fees are influenced by factors such as journal prestige, impact factor, scope of the journal, and the services provided by the publisher. Some OA journals have introduced waiver policies, which might reduce the costs for authors from poorly resourced countries, but often, the waiver process is opaque and routinely requires motivation for waivers. APCs may surpass the monthly salary of academics and researchers in Global South settings [4]. Regrettably, three-quarters of journals do not offer waivers for scientists from lower-income nations, perpetuating inequities in scientific knowledge [5].

Plan S, launched in 2018 by cOAlition S, an international consortium of research funding and performing organizations, seeks to make research publications openly available immediately upon publication in open access repositories, rather than behind paywalls or restricted access models [6]. To facilitate this, Plan S funders agreed to pay APCs to "gold" OA journals. This “transformative” arrangement will cease in 2024, with cOAlition S members intending to direct their efforts and funding to more innovative and community-led OA publishing initiatives thereafter.

OA journals are classified as gold, silver, bronze, green, diamond, or hybrid based on their fee structure. Gold OA journals publish articles with immediate reader access without restrictions following the payment of APCs by the authors. Green OA, also known as self-archiving, allows authors to share their research findings openly through a repository or website while complying with traditional publishing requirements. In 2018, most OA articles were published as "bronze", which allows the article to be read on the publisher’s website, but not copied or reused [7]. Diamond OA journals, available free to both readers and authors, are community-driven and academic-led and -owned publishing initiatives reliant on alternative funding models, and dependent on volunteer support. Hybrid journals allow authors the option to publish either by paying the APC (allowing OA) or by a subscription model with little to no publishing fees but restricted reader access.

An unintended consequence of the new publishing order may be an exacerbation of global inequities in scientific knowledge. Publishing in reputable but expensive OA journals means researchers in less-resourced settings divert funds from critical research needs, if indeed funds are available. Failure to publish in recognized publications or publishing behind paywalls fosters underrepresentation of diverse perspectives and hinders adding innovative solutions to the global discourse, simultaneously limiting career progression and future funding opportunities for the individual researcher.

Gold OA journals have attempted to address the criticism of their perceived high publishing costs by offering fee waivers or tiered pricing structures to authors from less resourced settings. Eligibility criteria for fee waivers include factors such as the World Bank's low-income economy classification, authors' institutional affiliation, and the quality and importance of the research. However, these have been criticized as shallow, window-dressing efforts designed to merely appease critics considering their extremely limited coverage. Current policies not only leave academics from middle-income countries, but also non-institutional-based and early career and retired researchers in better-resourced settings to fend for APC resources by themselves. Although government and funding agencies have been urged to finance these researchers' inputs, few do so. It has been estimated that including all accepted manuscripts from low and lower-middle-income countries in full waivers and offering 50% discounts to all in upper-middle-income economies would result in full waivers being granted to approximately 2% of authors and discounts to approximately 25% [8].

This earnings loss could easily be absorbed by an industry dominated by five publishers (Black and Wiley, Elsevier, SAGE, Springer Nature, and Taylor and Francis) with unusually high profit margins. The revenue generated by the scientific publishing industry in 2017 totaled over $19 billion [9]. Elsevier, for instance, boasted a profit margin approaching 40% with an upward trend curve [9]. APC models create pressure on publishers to favor quantity over quality, rewarding bulk publishing approaches benefiting the largest commercial publishers and promoting a thriving industry of predatory publishers. While OA journals defend their pricing policies by citing the prohibitive cost of publication, evidence suggests that this may be misleading. The publication costs for a representative scholarly article today are estimated to be around US$400 [10]. Additional non-publication items are said to make up the difference between publication costs and the final APC price charged [10]. It is understood that publishing is a business for many companies, thus profits are essential, but they need to be tempered by the reality that in LMICs the large profits are inhibitive from a researcher’s perspective, as publication of their research results is an essential to progress in an academic environment and for the improvement of health outcomes.

In this environment, what options exist to address the impending crisis? Two main kinds of solutions have emerged. One involves finding alternate mechanisms to fund OA publication costs, and the other proposes disrupting the current publishing system.

The primary mechanisms being proposed and adopted to address the funding gap are through (1) getting more journals to offer waivers and extending the coverage of those already offering these, (2) increased governmental interest and support, (3) universities, libraries, research institutions, and funders identifying and supporting different funding mechanisms, and (4) altering the structure of publication fee payment.

Strong political and civil society advocacy will be required to convince publishers to reduce profit margins. Funds generated by Plan S signatories could provide APC relief through waivers or discounts for disadvantaged researchers. Advocating for greater utilization of green OA through repositories such as arXiv.org is another viable option.

Regional and national research councils and international donors can alleviate the burden of publication costs through investment in open repositories and by offering dedicated grants and subsidies to cover APCs, as well as funding more open journals and high-quality publishing platforms. Similarly, research sponsors should allow grant applicants to include a line item to cover publishing fees. Governments could increase subsidies for universities to assist in covering full APCs. Universities, research institutions, and publishers could forge partnerships to negotiate lower or zero publication fees, similar to the agreement that exists between the National Institutes for Health (NIH) and Oxford journals [11].

Other suggestions offered include a shift from publication-based charging to process-based charging, researchers being able to choose to pay only for essential publication services and be exempted from covering marketing and other non-essential costs, and peer reviewers being rewarded through vouchers offered to their institutions that can be used to offset future publication costs. Another proposed option is the promotion of page charges rather than APCs, but this does not address the major issue of researchers having to bear the brunt of the publication costs and publisher profits. Although page charges may currently be lower than APCs there is nothing preventing a publisher from upping the page charges to the equivalent of APCs.

The disruption approach favors greater availability of author-free OA options through publication in diamond OA journals and devaluing the published article’s entrenched position as the sole research output of value, undermining movement towards an Open Science ecosystem. Relatively few papers are published in diamond OA journals, with many publishing fewer than 25 papers per year, often issued annually, and mostly belonging to social sciences and humanities. More than 70% of diamond OA journals are published by universities, around 15% by publishing companies, while the rest are published by societies, professional associations, or libraries [9]. A disruptive approach requires a shift in researchers’ mindset, recognizing and promoting the value of these journals, and actively seeking to publish in them. Simultaneously, the value of all research outputs—preprints, articles, data, peer review reports, etc—and grey literature should be emphasized and given equal recognition in academic evaluation systems.

While the OA movement has made strides in increasing access to scientific research, there remain critical challenges to overcome. The financial burden imposed by APCs presents a significant barrier to researchers, particularly those in less resourced settings, but so too does publishing in subscription-based journals. Efforts must be made to address these challenges through diverse funding mechanisms, including increased governmental support, collaboration between institutions and publishers, and exploring disruptive approaches to the current publishing system. By ensuring equitable access to publishing opportunities, we can foster global scientific collaboration, knowledge sharing, and improve healthcare outcomes in all settings.
